# Triptolide inhibits cell proliferation and tumorigenicity of human neuroblastoma cells

**DOI:** 10.3892/mmr.2014.2814

**Published:** 2014-10-29

**Authors:** XIAOMIN YAN, XIAO-XUE KE, HAILONG ZHAO, MENGYING HUANG, RENJIAN HU, HONGJUAN CUI

**Affiliations:** 1 State Key Laboratory of Silkworm Genome Biology, Southwest University, Chongqing 400716, P.R. China; 2 College of Pharmacy and Biological Engineering, Chongqing University of Technology, Chongqing 400050, P.R. China

**Keywords:** neuroblastoma, triptolide, cell proliferation, tumorigenicity, cell cycle arrest, apoptosis

## Abstract

Triptolide is a diterpene triepoxide, extracted from the Chinese herb *Tripterygium wilfordii* Hook F, which has been shown to have antitumor activity in a number of cancers. Neuroblastoma is an aggressive extracranial pediatric solid tumor, with significant chemotherapeutic resistance. In this study, triptolide was hypothesized to be a potential therapeutic agent for neuroblastoma. The effects of triptolide on neuroblastoma cell growth and tumor development were investigated. Cell growth and proliferation were evaluated using a cell counting kit-8 assay and a 5-bromo-2-deoxyuridine staining assay. Cell cycle and apoptosis were detected by flow cytometry. Reverse transcription-quantitative polymerase chain reaction was conducted to detect the expression levels of the apoptosis-associated proteins, caspase-3 and caspase-9. The tumorigenicity of neuroblastoma cells was assessed by a soft agar clonogenic assay and an *in vivo* tumorigenic assay. The results demonstrated that exposure of BE(2)-C human neuroblastoma cells to triptolide resulted in a reduction in cell growth and proliferation, and the induction of cell death and apoptosis, together with cell cycle arrest in the S phase. A soft agar assay indicated that triptolide inhibited the colony-forming ability of BE(2)-C neuroblastoma cells. The xenograft experiment showed that triptolide significantly reduced tumor growth and development *in vivo*. The data suggested that this Chinese herb may be a potential novel chemotherapeutic agent for neuroblastoma.

## Introduction

Neuroblastoma is a common extracranial pediatric solid tumor, accounting for up to 10% of pediatric cancers and ultimately resulting in 15% of cancer-related mortality in children (1,2). Histologically, neuroblastoma is a heterogeneous group of tumors ranging from benign changes in sympathetic neurons to tumors that cause critical illness as a result of extensive invasion and metastasis (3–5). The clinical presentation of neuroblastoma is variable and advanced cases are often found to be highly resistant to conventional treatment modalities based on surgery, chemotherapy and radiotherapy (6). Therefore, recent studies have primarily focused on these particularly aggressive cases, with the goal of identifying additional therapeutic options (7).

Natural compounds extracted from herbs, such as Taxol, have been widely used in cancer therapy (8). A number of active compounds isolated from Chinese herbs have been shown to have antitumor properties (9,10). Thus, traditional Chinese medicine provides an important additional option for the development of novel cancer treatments. Triptolide is a diterpene triepoxide and is extracted from the Chinese herb *Tripterygium wilfordii* Hook F, which has been used to treat inflammation and autoimmune diseases in Chinese medicine (11,12). Recently, evidence has shown that triptolide has a potent immunosuppressive effect and antineoplastic activity in certain types of cancer, including breast cancer, pancreatic cancer, melanoma and prostate cancer (13–16). In addition, triptolide has been shown to exert its antitumor properties through induction of apoptosis and inhibition of cell proliferation, angiogenesis, cell invasion and metastasis (17–20). This study investigated the effects of triptolide on malignant neuroblastoma cell growth and cell proliferation with the aim of providing evidence that may support the use of triptolide as a novel drug for the treatment of neuroblastoma.

## Materials and methods

### Cell culture

BE(2)-C neuroblastoma cells were obtained from the American Type Culture Collection (Manassus, VA, USA), and was cultured in a 1:1 mixture of Dulbecco’s modified Eagle’s medium and Ham’s nutrient mixture F12 (DMEM/F-12; Invitrogen, Carlsbad, CA, USA) plus 10% fetal bovine serum (Invitrogen) and 1% penicillin and streptomycin (Invitrogen), and was incubated at 37°C in a 5% CO_2_ humidified incubator. Purified triptolide (>98%) was purchased from Sigma-Aldrich (T3652; St. Louis, MO, USA), which was dissolved in dimethyl sulfoxide (DMSO; Sigma-Aldrich) at a stock concentration of 50 mM and stored at 4°C.

### Cell growth and viability assays

BE(2)-C cells grown in 96-well culture plates were treated either with various doses of triptolide (5, 10, 25, 50 or 100 nM), or DMSO. The cell growth rate was analyzed with the cell counting kit-8 (CCK-8) growth assay after 24 h culture. Briefly, cells in each well were incubated with 10 *μ*l CCK-8 reagent at 37°C for 2 h. The optical density was measured at a wavelength of 450 nm using a microplate reader (Model 550, Bio-Rad, Hercules, CA, USA). In addition, BE(2)-C cells were treated with 25 or 50 nM for 24 h, photographed using an Olympus 1X71 (Olympus Corporation, Tokyo, Japan) and counted with a TC10^™^ Automated Cell Counter (Bio-Rad).

### 5-Bromo-2-deoxyuridine (BrdU) staining assay

For BrdU immunofluorescent staining, cells were grown on coverslips. After treatment with 25 or 50 nM triptolide for 24 h, cells were incubated with 10 *μ*g/ml BrdU (Sigma) for 30 min, then washed with phosphate-buffered saline (PBS) and fixed in 4% paraformaldehyde for 20 min. Subsequently, cells were treated with 1 mol/L HCl, and blocked with 10% goat serum for 1 h, followed by a monoclonal rat primary antibody against BrdU (1:200, ab6326, Abcam, Cambridge, MA, USA) for 1 h and Alexa FluorR^®^ 594 goat anti-rat IgG secondary antibody, (H+L; Invitrogen). DAPI (300 nM) was used for nuclear staining, after which the percentage BrdU uptake in 10 microscopic fields was calculated (Nikon 80i, Nikon Corporation, Tokyo, Japan).

### Cell cycle assay

After treatment with triptolide (25 nM) for 24 h, cells were collected by centrifugation at 211 × g for 5 min, washed with ice-cold PBS, fixed with 70% ethanol, stained with 20 *μ*g/ml propidium iodide (Invitrogen) and analyzed by flow cytometry (BD FACSVerse™, BD BioSciences, Franklin Lakes, NJ, USA). The data were analyzed with CellQuest Pro software, version 5.0 (BD BioSciences).

### Cell death and apoptosis assays

Cells were either untreated or treated with triptolide. DMSO was used as control. After 24 h treatment, adherent and floating cells were pooled, collected by centrifugation at 211 × g for 5 min, and washed once with ice-cold PBS. The cell death rate was detected with 0.2% trypan blue dye (Bio-Rad). Apoptotic cells were determined by the Annexin V-fluorescein isothiocyanate (FITC) kit (Sigma-Aldrich), using flow cytometry according to the manufacturer’s instructions.

### Reverse transcription-quantitative polymerase chain reactions (RT-qPCR) assay

After treatment with triptolide for 24 h, cells were harvested and lysed with TRIzol (Invitrogen) for total RNA purification. RNA was reverse transcribed into cDNA using M-MLV reverse transcriptase (Promega Corporation, Madison, WI, USA). The caspase-3 and caspase-9 mRNA transcripts were determined using the SYBRR Green PCR Master mix (Takara Bio, Inc., Shiga, Japan) by RT-qPCR. RT-qPCR reactions in triplicate were conducted using the OneStep plus7500 real-time PCR system (Bio-Rad). The individual values were normalized to that of the GAPDH control. Primer sequences were as follows: Forward: 5′-AGCGAATCAATGGACTCTGGA-3′ and reverse: 5′-CTGAATGTTTCCCTGAGGTTTG-3′ for caspase-3, forward: 5′-GCTCTTCCTTTGTTCATCTCC-3′ and reverse: 5′-CATCTGGCTCGGGGTTACTGC-3′ for caspase-9, and forward: 5′-ACGGATTTGGTCGTATTGGG-3′ and reverse: 5′-TCCTGGAAGATGGTGATGGG-3′ for GAPDH.

### Soft agar clonogenic assay

Cells (1×10^3^) were mixed in 0.3% Noble agar in a growth medium containing vehicle or triptolide, and plated into six-well plates containing a solidified bottom layer (0.6% Noble agar in the same growth medium). Colonies were photographed after 14 days (Olympus 1X71) and cell numbers were counted from at least five randomly selected fields.

### In vivo tumorigenic assay

Six female non-obese diabetic (NOD)/severe combined immunodeficiency (SCID) mice (4 weeks old) were used and maintained under specific pathogen-free conditions. Cells (1×10^6^) were suspended in 200 *μ*l serum-free DMEM/F12, and injected subcutaneously into both flanks. After tumor formation, mice were divided randomly into treatment and control groups. Mice in the treatment group were injected with triptolide at 0.4 mg/kg daily for seven days (21), while control mice were injected with DMSO. Tumor size was measured using calipers and tumor volume was calculated using the formula 4/3πr^3^, where r is the radius of the tumor. Mouse body weight was monitored daily. Three weeks after tumor growth, xenograft tumors were removed and weighed immediately after the mice were sacrificed by cervical dislocation. All animal experiments were approved by the Institutional Animal Care and Use Committee of Southwest University (Chonqing, China).

### Statistical analysis

All observations were confirmed by at least three independent experiments. Quantitative data are expressed as the mean ± standard deviation. Two-tailed Student’s t-test was performed for paired samples using GraphPad Prism version 6.0 (GraphPad Software, Inc., La Jolla, CA, USA). P<0.05 was considered to indicate a statistically significant difference.

## Results

### Triptolide inhibits neuroblastoma cell growth and viability

**Figure 1:**
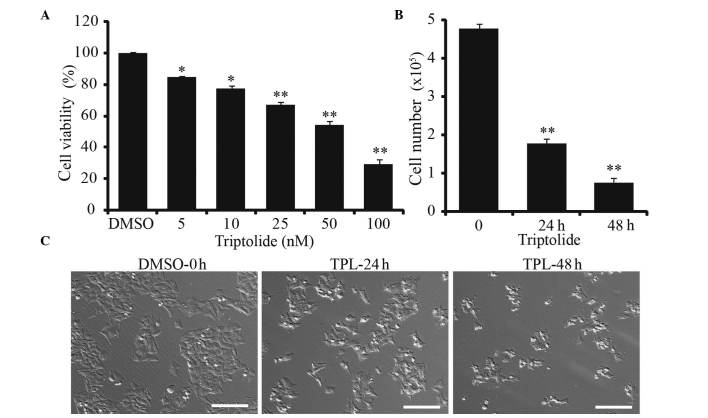
TPL inhibited neuroblastoma cell growth and viability. (A) BE(2)-C cells were treated with 5, 10, 25, 50 and 100 nM TPL for 24 h. DMSO was used as a control. Cell viability was assessed by a CCK-8 assay. (B) BE(2)-C cell numbers from panel C were counted using the TC10™ Automated Cell Counter.(C) Morphologic examination of BE (2)-C cells treated with 25 nM triptolide for the indicated times (0, 24 or 48 h). Scale bar, 100 *μ*m. Each value represents the average obtained from three independent experiments. Data are presented as the mean ± standard deviation. ^*^P<0.05 and ^**^P<0.01, compared with control. CCK-8, cell counting kit-8; TPL, triptolide; DMSO, dimethyl sufoxide.

**Figure 2:**
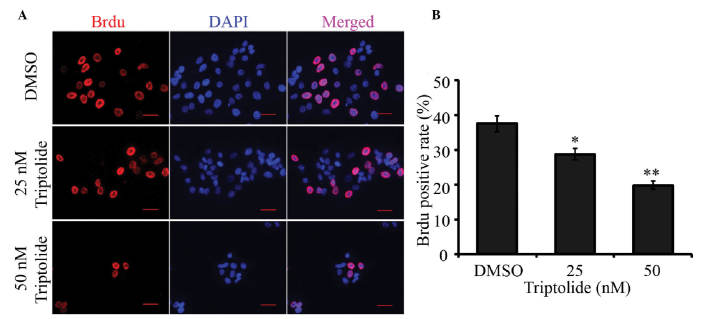
BrdU immunofluorescence staining assay. (A) Immunofluorescence staining of Brdu in BE(2)-C cells treated with triptolide (25 and 50 nM) for 24 h. Scale bars, 25 *μ*m. (B) The percentage of BrdU positive cells from panel A was calculated. Each value represents the average obtained from three independent experiments. Data are presented as the mean ± standard deviation. ^*^P<0.05 and ^**^P<0.01, compared with control. BrdU, 5-bromo-2-deoxyuridine; DMSO, dimethyl sulfoxide.

BE(2)-C cells were treated with increasing doses of triptolide for 24 h. A concentration-dependent response to triptolide in the BE(2)-C cells was observed. As shown in Fig. 1A, triptolide inhibited cell growth even at a low dose of 5 nM. The cell viability was significantly reduced to 50% at 50 nM of triptolide. Triptolide also inhibited cell growth in a time dependent manner (Fig. 1B and C). Moreover, immunofluorescent staining using a BrdU label confirmed that triptolide markedly inhibited cell proliferation (Fig. 2A and B).

### Triptolide induces neuroblastoma cell cycle arrest and apoptosis

**Figure 3:**
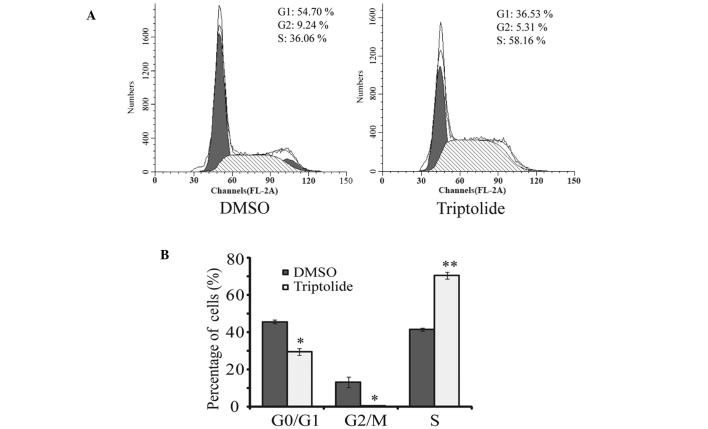
Triptolide induced neuroblastoma cell cycle arrest in the S phase. (A) BE(2)-C cells were either treated with DMSO or 25 nM triptolide for 24 h. Cells were harvested, fixed with ethanol and stained with propidium iodide. DNA content was determined by flow cytometry. (B) Analysis of cell cycle phase percentage in BE(2)-C cells from panel A. Each column represents the average obtained from three independent experiments. Data are presented as the mean ± standard deviation. ^*^P<0.05 and ^**^P<0.01, compared with control. DMSO, dimethyl sulfoxide.

The effect of triptolide on cell cycle was investigated. It was found that the percentage of cells in S phase increased from 36.06 to 58.16% (Fig. 3A and B). This result suggests that triptolide induces cell cycle arrest in the S phase, which may contribute to inhibition of cell proliferation.

**Figure 4:**
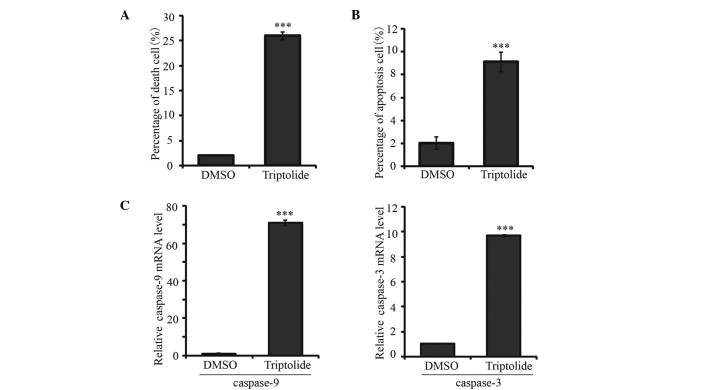
Triptolide induced neuroblastoma cell apoptosis through caspase-9/caspase-3 pathway activation. Analysis of the percentage of (A) dead and (B) apoptotic BE(2)-C cells. BE(2)-C cells were treated with 25nM triptolide for 24 h, and cell death and apoptosis were determined by trypan blue dye and Annexin V-fluorescein isothiocyanate kit, respectively. DMSO was used as a control. (C) mRNA expression levels of caspase-3 and caspase-9 in BE(2)-C cells treated with DMSO or triptolide were determined by RT-qPCR analysis. Data represent the average obtained from three independent experiments. Data are presented as the mean ± standard deviation. ^*^P<0.05 and ^**^P<0.01, compared with control. RT-qPCR, reverse transcription-quantitative polymerase chain reaction; DMSO, dimethyl sulfoxide.

It was also observed that exposure of BE(2)-C cells to triptolide could induce cell death and apoptosis. Triptolide significantly increased cell death from 1.88 in the control group to 25.9% in the triptolide 25 nM group (P<0.001; Fig. 4A). The apoptosis rate was also increased after treatment with 25 nM triptolide for 24 h (Fig. 4B). As shown in Fig. 4C and D, following triptolide treatment mRNA expression levels of caspase-9 and caspase-3 were increased 70.9 and 9.7 fold, respectively, compared with control. These results indicate that triptolide induces cell death and apoptosis through caspase-9 and caspase-3 activation.

### Triptolide suppresses neuroblastoma cell colony formation in vitro and tumorigenicity in vivo

**Figure 5:**
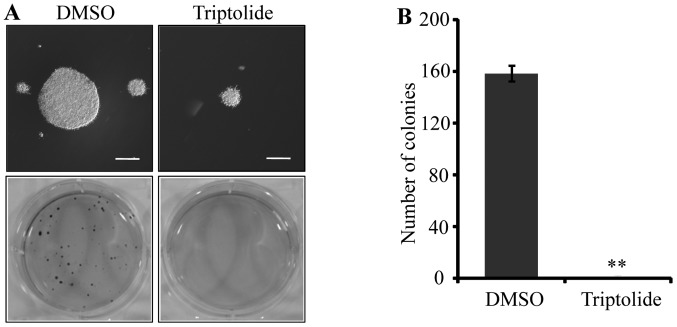
Triptolide suppressed BE (2)-C cells colony-forming capability. (A) Soft agar clonogenic assay of BE(2)-C cells was performed after treatment with triptolide. After 14 days of culture, images of colonies (larger than 1.0 mm or containing more than 150 cells) were captured. Scale bars, 50 *μ*m. (B) Analysis of colony formation numbers from panel A was performed. Cells were counted from at least five randomly selected fields. Data are presented as the mean ± standard deviation. ^*^P<0.05 and ^**^P<0.01, compared with control. DMSO, dimethyl sulfoxide.

**Figure 6:**
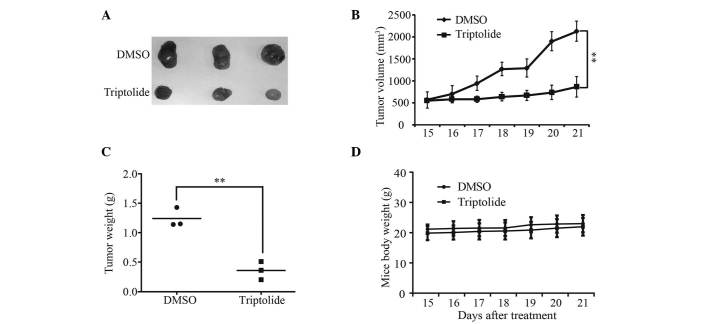
Triptolide inhibited tumor growth and development in the xenograft model. BE(2)-C cells (1×10^6^) were injected subcutaneously into the flanks of NOD/SCID mice. After tumor formation (approximately two weeks), mice were injected with DMSO or triptolide (0.4 mg/kg) daily for seven days. (A) Images of tumors dissected from NOD/SCID mice after treatment with triptolide or DMSO. (B) Xenograft tumors were measured daily, after treatment with triptolide or DMSO, by calipers. (C) Scatter plot of xenograft tumor weight with horizontal lines indicated the mean in each group. (D) The average body weight of NOD/SCID mice was monitored daily after treatment with triptolide or DMSO. Data are presented as the mean ± standard deviation. ^*^P<0.05 and ^**^P<0.01, compared with control. DMSO, dimethyl sulfoxide; NOD mice, non-obese diabetic mice; SCID mice, severe combined immunodeficiency mice.

The role of triptolide in neuroblastoma tumorigenesis was examined. BE(2)-C cells treated with 25 nM triptolide gave rise to smaller and and sparser colonies in soft agar, compared with cells treated with DMSO (Fig. 5A and B). The xenograft study in NOD/SCID mice showed that the volume and weight of xenograft tumors in the triptolide treatment group were lower than those in the DMSO group (Fig. 6). These data indicate that triptolide may inhibit neuroblastoma cell self-renewal and tumorigenesis. In addition, there was no significant difference in mouse body weight after triptolide treatment (Fig. 6D), which suggests that the administered dose of triptolide may have minimal toxic side effects.

## Discussion

Recently, Chinese herbs have attracted attention from researchers worldwide due to their potential efficacy in the treatment of a number of diseases (22,23). A large number of active compounds have been extracted from Chinese herbs. T*ripterygium wilfordii* Hook F has been used in traditional Chinese medicine for centuries for the treatment of fever, chills, carbuncles and edema (24,25). The diterpenoid epoxide triptolide is one of the two main bioactive components of *Tripterygium wilfordii* Hook F, which exhibits antitumor activity (26,27). However, there is little data regarding the efficacy of triptolide against neuroblastoma cells. This study aimed to investigate the effect of triptolide on neuroblastoma cell growth and tumor development, with the aim of providing more information for the development of novel neuroblastoma treatments.

The current study demonstrated that triptolide not only induced neuroblastoma cell death and apoptosis via caspase-9/caspase-3 pathway activation, but also inhibited cell growth and viability by inducing cell cycle arrest at the S phase. Furthermore, the results showed that triptolide inhibited neuroblastoma cell colony-forming capability *in vitro* and tumor progression *in vivo*. In conclusion, triptolide may be a potent natural candidate for neuroblastoma treatment.

## References

[1] Castleberry RP, Pritchard J, Ambros P (1997). The International Neuroblastoma Risk Groups (INRG): a preliminary report. Eur J Cancer.

[2] Li T, Wang L, Ke XX (2012). DNA-damaging drug-induced apoptosis sensitized by N-myc in neuroblastoma cells. Cell Biol Int.

[3] Shimada H, Ambros IM, Dehner LP (1999). The International Neuroblastoma Pathology Classification (the Shimada system). Cancer.

[4] Brodeur GM (2003). Neuroblastoma: biological insights into a clinical enigma. Nat Rev Cancer.

[5] Cui H, Ma J, Ding J (2006). Bmi-1 regulates the differentiation and clonogenic self-renewal of I-type neuroblastoma cells in a concentration-dependent manner. J Biol Chem.

[6] Cheung NK, Dyer MA (2013). Neuroblastoma: developmental biology, cancer genomics and immunotherapy. Nat Rev Cancer.

[7] Morgenstern DA, Baruchel S, Irwin MS (2013). Current and future strategies for relapsed neuroblastoma: challenges on the road to precision therapy. J Pediatr Hematol Oncol.

[8] Camirand A, Fadhil I, Luco AL (2013). Enhancement of taxol, doxorubicin and zoledronate anti-proliferation action on triple-negative breast cancer cells by a PTHrP blocking monoclonal antibody. Am J Cancer Res.

[9] Jia L, Ma S, Hou X (2013). The synergistic effects of traditional Chinese herbs and radiotherapy for cancer treatment. Oncol Lett.

[10] Kavandi L, Lee LR, Bokhari AA (2013). The Chinese herbs Scutellaria baicalensis and Fritillaria cirrhosa target NFκB to inhibit proliferation of ovarian and endometrial cancer cells. Mol Carcinog.

[11] Hailong G, Yujie Z, Hanying M (2011). Effectiveness of triptolide-coated stent on decreasing inflammation and attenuation of intimal hyperplasia in a pig after coronary angioplasty. Angiology.

[12] Wu R, Li Y, Guo Z (2013). Triptolide ameliorates ileocolonic anastomosis inflammation in IL-10 deficient mice by mechanism involving suppression of miR-155/SHIP-1 signaling pathway. Mol Immunol.

[13] Owa C, Messina ME, Halaby R (2013). Triptolide induces lysosomal-mediated programmed cell death in MCF-7 breast cancer cells. Int J Womens Health.

[14] Banerjee S, Sangwan V, McGinn O (2013). Triptolide-induced cell death in pancreatic cancer is mediated by O-GlcNAc modification of transcription factor Sp1. J Biol Chem.

[15] Chueh FS, Chen YL, Hsu SC (2013). Triptolide induced DNA damage in A375.S2 human malignant melanoma cells is mediated via reduction of DNA repair genes. Oncol Rep.

[16] Chen YW, Lin GJ, Hueng DY (2014). Enhanced anti-tumor activity of triptolide in combination with irradiation for the treatment of oral cancer. Planta Med.

[17] Wang XF, Zhao YB, Wu Q (2014). Triptolide induces apoptosis in endometrial cancer via a p53-independent mitochondrial pathway. Mol Med Rep.

[18] Krizanova O, Markova J, Pacak K (2014). Triptolide induces apoptosis through the SERCA 3 upregulation in PC12 cells. Gen Physiol Biophys.

[19] Johnson SM, Wang X, Evers BM (2011). Triptolide inhibits proliferation and migration of colon cancer cells by inhibition of cell cycle regulators and cytokine receptors. J Surg Res.

[20] Ma JX, Sun YL, Wang YQ (2013). Triptolide induces apoptosis and inhibits the growth and angiogenesis of human pancreatic cancer cells by downregulating COX-2 and VEGF. Oncol Res.

[21] Antonoff MB, Chugh R, Borja-Cacho D (2009). Triptolide therapy for neuroblastoma decreases cell viability in vitro and inhibits tumor growth in vivo. Surgery.

[22] Wan YG, Che XY, Sun W (2014). Low-dose of multi-glycoside of Tripterygium wilfordii Hook. f., a natural regulator of TGF-β1/Smad signaling activity improves adriamycin-induced glomerulosclerosis in vivo. J Ethnopharmacol.

[23] Ge Y, Xie H, Li S (2013). Treatment of diabetic nephropathy with Tripterygium wilfordii Hook F extract: a prospective, randomized, controlled clinical trial. J Transl Med.

[24] Chen Y, Gong Z, Chen X (2013). Tripterygium wilfordii Hook F (a traditional Chinese medicine) for primary nephrotic syndrome. Cochrane Database Syst Rev.

[25] Helmstädter A (2013). Tripterygium wilfordii Hook. f. - how a traditional Taiwanese medicinal plant found its way to the West. Pharmazie.

[26] Huang W, He T, Chai C (2012). Triptolide inhibits the proliferation of prostate cancer cells and down-regulates SUMO-specific protease 1 expression. PLoS One.

[27] Liu Z, Ma L, Wen ZS (2014). Cancerous inhibitor of PP2A is targeted by natural compound celastrol for degradation in non-small-cell lung cancer. Carcinogenesis.

